# First Reproduction of *Octopus mimus* Under Controlled Aquaculture Conditions in Southern Peru: Conditioning, Water Quality, and Morphometric Evaluation of Breeders

**DOI:** 10.3390/ani16040645

**Published:** 2026-02-17

**Authors:** Calixto Quispe-Pilco, Khiara Aliyah Bet Moreno-Salazar-Calderón, Freddy Walter Delgado-Cabrera, Fredy Esfrayn Tapia-Alave, Juan Zenón Resurrección-Huertas, Cintia Pamela Fernández-Cárdenas, Jordan I. Huanacuni

**Affiliations:** 1 Escuela Profesional de Ingeniería Pesquera, Facultad de Ciencias Agropecuarias, Universidad Nacional Jorge Basadre Grohmann, Tacna 23004, Peru; cquispe@unjbg.edu.pe (C.Q.-P.); kmorenos@unjbg.edu.pe (K.A.B.M.-S.-C.); fdelgadoc@unjbg.edu.pe (F.W.D.-C.); ftapiaa@unjbg.edu.pe (F.E.T.-A.); cfernandezc@unjbg.edu.pe (C.P.F.-C.); 2 Grupo de Investigación Acuicultura Sostenible, Universidad Nacional Jorge Basadre Grohmann, Tacna 23000, Peru; 3 Escuela Profesional de Ingeniería Acuícola, Facultad de Ingeniería Pesquera, Universidad Nacional José Faustino Sánchez Carrión, Huacho 15135, Peru; pezsaulo@gmail.com; 4 Campus Tacna, Universidad Tecnológica del Perú, Tacna 23004, Peru

**Keywords:** adaptability, cephalopods, environmental conditions, aquaculture diversification, reproduction

## Abstract

*Octopus mimus aquaculture* has gained relevance owing to its commercial potential; however, optimization of its reproductive and growth conditions in captivity requires further study. The objective of this study was to evaluate the reproductive conditioning, morphometric growth, and water quality of this species in a controlled system. To do this, adult *O. mimus* were selected and kept in circular tanks with controlled temperature, dissolved oxygen, pH, and salinity. Weight, total length (TL), mantle length (ML), and arm length (AL) measurements were made, in addition to monitoring seawater quality throughout the conditioning cycle. Paralarvae hatching productions were recorded, and Fulton’s condition index (*k*) was calculated to assess octopus health. The results showed that larger females produced more paralarvae, and that temperature significantly influenced the incubation period. The morphometric relationships between TL, ML, and LA were highly correlated, particularly in females. The seawater quality, with temperatures close to 22 °C, dissolved oxygen between 7.8 and 9.2 mg/L, 7.1–8.0 pH, and salinity around 35.3 PSU, was optimal for the welfare and reproduction of octopuses. This study provides keys to the effective management of *O. mimus* in aquaculture, with an emphasis on the importance of proper water quality management and the selection of larger broodstock.

## Simple Summary

This study evaluated the reproductive conditioning, morphometric growth, and water quality of *Octopus mimus* in a controlled aquaculture system. Adult octopuses were kept in circular tanks with controlled temperature, dissolved oxygen, pH, and salinity levels. Larger females produced more paralarvae, and temperature significantly affected the incubation time. High correlations were found between total length (TL), mantle length (ML), and arm length (AL), particularly in females. Optimal seawater conditions (22 °C, 7.8–9.2 mg/L dissolved oxygen, and 35.3 PSU salinity) supported octopus welfare and reproduction, highlighting the importance of water quality management and selecting larger breeders.

## 1. Introduction

Cephalopod aquaculture has gained relevance in recent decades owing to the rapid growth, high feed conversion efficiency, behavioral plasticity, and capacity to reproduce in captivity of several cephalopod species, making them attractive candidates for commercial production [1,2]. Among cephalopods, octopuses have received particular attention because of their high market value and biological suitability for farming purposes. However, the successful implementation of octopus aquaculture on an industrial scale remains limited by unresolved biological and technical constraints [3,4,5]. A central challenge in octopus aquaculture is associated with species exhibiting a merobenthic life cycle, characterized by a planktonic paralarval phase, followed by benthic juvenile and adult stages [4]. Although adult octopuses can be readily maintained in captivity and spawning can be achieved under controlled conditions, the survival and successful development of planktonic paralarvae remain the main bottlenecks for closing the life cycle [2,4]. In contrast to large-egg octopus species, which produce benthic hatchlings with higher initial survival, small-egg species such as *Octopus mimus* produce planktonic paralarvae that require highly specific nutritional and environmental conditions, greatly increasing the complexity of captive reproduction. Paralarvae have complex nutritional requirements, high metabolic demands, and strong sensitivity to environmental conditions, resulting in high mortality rates in captivity [6]. Consequently, documenting reproductive output and paralarval production under controlled conditions is a critical step toward advancing octopus aquaculture.

Most advances in octopus aquaculture research have been achieved using *O. vulgaris*, which is considered the best-studied species in this field [3,4,7]. Studies on *O. vulgaris* have demonstrated that temperature, diet quality, broodstock conditioning, and water quality are key determinants of fecundity, egg quality, and paralarval viability [2,3,7]. Despite these advances, the complete closure of *O. vulgaris* under commercial-scale conditions remains challenging, underscoring the complexity of octopus reproduction and early life stages [4].

In contrast, *O. mimus* (Gould, 1852) has received comparatively less attention, despite exhibiting biological traits that may be advantageous for aquaculture. This species inhabits the southern Pacific coast from northern Peru (Tumbes) to central Chile (Bahía San Vicente) [8]. Recent morphological and molecular evidence suggests that *Octopus hubbsorum* may be a junior synonym of *O. mimus*, potentially expanding its recognized distribution along the Eastern Pacific coast [9]. This wide geographic distribution reflects a high degree of ecological plasticity, which may facilitate adaptation to diverse environmental conditions in captivity [10]. *O. mimus* is of high economic importance in Peru and Chile, where it is traditionally exploited by artisanal fisheries and supports local consumption and international export markets [11]. Compared with *O. vulgaris*, *O. mimus* presents a shorter life cycle, rapid growth, and apparent tolerance to a variety of diets, characteristics that may enable more flexible and accessible feeding protocols in aquaculture systems [6,10]. However, despite these favorable traits, knowledge of their reproductive performance under controlled conditions remains limited. Most previous studies on *O. mimus* have focused on its biology and ecology in the wild, including diet, growth patterns, morphometric relationships, and size at sexual maturity [10,11,12]. These studies have shown that morphometric and size-to-weight relationships vary across geographical regions and environmental conditions [11]. For instance, female *O. mimus* reach sexual maturity at a mantle length of approximately 14.3 cm, which has been used to propose minimum catch sizes for fishery management [11]. Nevertheless, systematic studies addressing captive reproduction, fecundity, and spawning success in relation to water quality and dietary factors are scarce [2,8]. Previous research has identified temperature and feeding regimes as key factors influencing growth and reproduction in *O. mimus* [1,13], similar to the patterns observed in *O. vulgaris* [3,7]. However, species-specific differences in physiology, ecology, and reproductive strategies indicate that culture protocols developed for *O. vulgaris* cannot be directly transferred to *O. mimus* without targeted investigations [2]. Rather than being mutually exclusive, *O. vulgaris* and *O. mimus* represent complementary models for octopus aquaculture research. *O. vulgaris* remains the primary reference species due to its extensive experimental background and technological development, whereas *O. mimus* exhibits biological traits, such as a shorter life cycle, rapid growth, and broad ecological tolerance, that may facilitate adaptation to diverse culture conditions, particularly in the Southeast Pacific. Consequently, both species are suitable candidates for aquaculture, albeit with different research priorities and region-specific applications that require further investigation.

Therefore, expanding the baseline knowledge of the reproductive biology of *O. mimus* under controlled conditions is essential to advance its aquaculture potential. The present study did not aim to experimentally optimize reproductive conditions but rather to document baseline reproductive performance, morphometric relationships, and environmental parameters, such as temperature, salinity, dissolved oxygen, and pH, under which successful spawning occurred in captivity in Peru. Such information constitutes a necessary step toward closing the life cycle of *O. mimus*, supporting aquaculture diversification, reducing fishing pressure on wild populations, and contributing to the development of sustainable and potentially restorative aquaculture initiatives in the Southeast Pacific [6,10].

## 2. Materials and Methods

### 2.1. Collection Area

Thirty-three *O. mimus* specimens were collected from the rocky beaches of Boca del Río, Tacna, in the southern coastal region of Peru during the spring of 2023 (October and November). The collection was carried out manually using freediving, a technique that allowed efficient and careful collection without causing harm to the specimens themselves. The octopuses were immediately placed in 20 L plastic buckets filled with fresh seawater to reduce stress and prevent physical injury during transport. Once the specimens arrived at the Specialized Laboratory of Mariculture and Fisheries, located at the Vila Vila Village Center, Sama District, Tacna, they were placed in conditioning tanks to initiate the reproductive management process.

**Figure 1:**
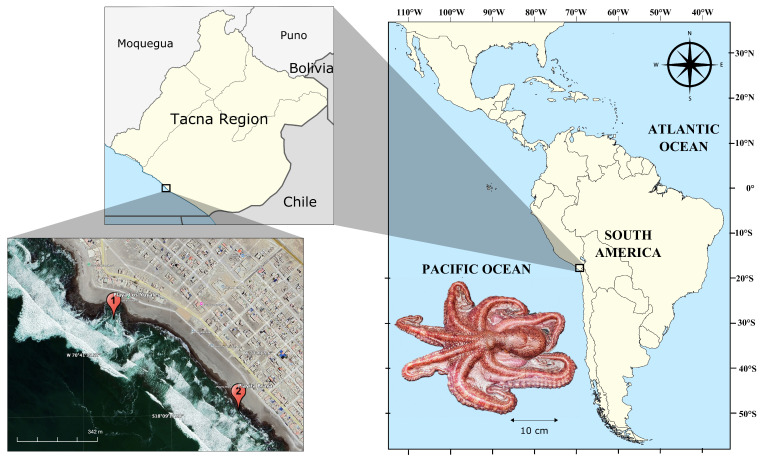
Location of the *Octopus mimus* collection areas on Los Yauyales and Playita Brava beaches in Tacna, Peru.

The collection areas were Los Yauyales Beach (18°9′3.64″ S, 70°41′31.04″ W) and Playita Brava (18°9′16.65″ S, 70°41′12.78″ W). These locations were chosen based on their favorable ecological conditions for *O. mimus*, with rocky substrates and a stable marine environment that provided optimal habitats for the species. Figure 1 shows the geographic locations of the collection areas.

### 2.2. Broodstock Conditioning

The broodstock consisted of 33 *O*. *mimus* individuals, including 25 females and 8 males, all of which were maintained in captivity throughout the study period. Of the 25 females maintained under captive conditions, seven successfully spawned during the evaluation period, whereas the remaining females did not exhibit spawning activity. All individuals were included in the morphometric analyses, whereas reproductive performance parameters were derived exclusively from females that successfully spawned during the evaluation period.

**Figure 2:**
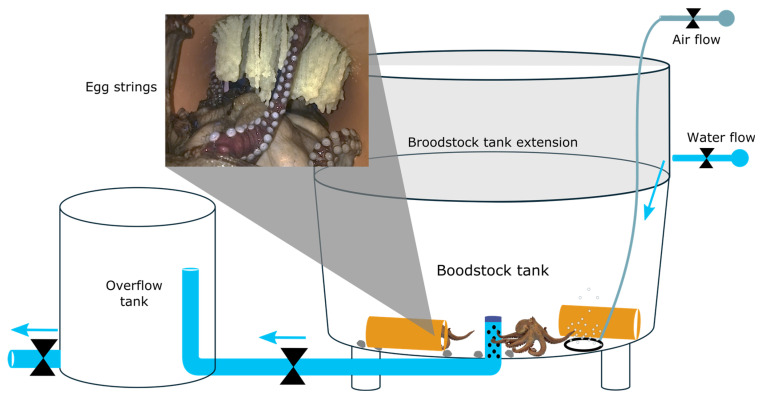
Circular tank culture system used for conditioning *Octopus mimus*.

Conditioning was conducted in a semi-open flow-through aquaculture system using circular fiberglass tanks (1.6 m diameter, 0.55 m depth). The system was supplied with seawater sourced directly from the adjacent coastal environment, which was mechanically pre-filtered prior to entering the tanks. The system did not operate as a closed recirculating aquaculture system (RAS). Water renewal was continuous, providing an exchange rate equivalent to approximately five complete tank volumes per day, ensuring stable physicochemical conditions and effective waste removal rather than discrete batch water changes. Stocking density was calculated at the system level (approximately 2–5 m^2^ per individual) to ensure low visual and chemical interaction among tanks and to minimize chronic stress. Octopuses were housed individually in separate shelters under normal conditions. Shelters consisting of PVC pipes (25 cm diameter, 50 cm length) and natural rocks collected from the marine environment. These shelters allowed expression of natural refuge-seeking behavior, reduced territorial aggression and minimized unnecessary mortality. Controlled and temporary cohabitation between males and females was allowed exclusively during specific mating periods, after which individuals were separated again (Figure 2).

The acclimation period lasted 24 days, during which water quality parameters—temperature (19.15–25.3 °C), dissolved oxygen (7.88–9.11 mg L^−1^), and pH (7.14–7.99)—were continuously monitored to ensure conditions suitable for *O. mimus*. Nitrogenous compounds were not systematically quantified, representing a limitation of the study. Broodstock were fed a varied diet of fresh, non-live prey, including crustaceans (*Ocypode gaudichaudii*, *Grapsus grapsus*, *Cancer porteri*), mollusks (*Gari solida*, *Aulacomya atra*), and fish (*Sarda chiliensis*, *Chirodactylus variegatus*). Food was provided ad libitum, and uneaten material was removed the following day to prevent organic matter decomposition and deterioration of water quality, which is critical for maintaining suitable captive conditions for cephalopods. Nitrogenous compounds (ammonia, nitrite, and nitrate) were not systematically quantified during the observational period, which represents a limitation of the present study. However, the semi-open flow-through system with continuous seawater renewal likely reduced the accumulation of metabolic wastes.

### 2.3. Spawning

Female *O. mimus* released their eggs in clusters that adhered to PVC pipes placed in their shelters inside conditioning tanks. During spawning and the incubation period, females did not consume food, which is typical behavior in cephalopods, as females prioritize the protection of their eggs and incubation over feeding.

Controlled cohabitation between males and females was allowed during the conditioning period to facilitate natural mating under captive conditions. Mating behavior was directly observed during these controlled cohabitation periods. Individuals were housed at a 1:1 male-to-female ratio within the same tank for periods ranging from several hours up to 48 h, depending on behavioral compatibility. Only one male and one female were present per tank during each cohabitation event. Typical mating behaviors, including arm extension and hectocotylus insertion, were observed. After mating, individuals were separated and returned to individual housing. Fertilization was therefore assumed to have occurred in captivity prior to spawning. Of the 25 females maintained, seven successfully spawned. The remaining females did not spawn during the study period and did not show external signs of senescence, such as reduced feeding or lethargy. The absence of spawning in some females, despite stable water quality and lack of overt stress indicators, suggests that additional intrinsic factors such as reproductive maturity, prior mating history, or individual physiological condition may have influenced spawning success. Because this study followed an observational design without hormonal, nutritional, or environmental manipulation, the factors limiting spawning in these females could not be determined. Once the eggs began to hatch, the number of paralarvae was counted. The newly hatched paralarvae were carefully collected and placed in a 1-L beaker. To determine the total number of paralarvae, a volumetric technique was used, followed by an accurate count using a stereoscope. This methodology allowed us to obtain an accurate estimate of the number of paralarvae produced by each female, which is essential for evaluating the reproductive success and efficiency of the incubation process under controlled conditions.

### 2.4. Morphometric Measurement and Condition Factor

To evaluate the physical condition and growth of the octopuses, morphometric measurements, including total length (TL), mantle length (ML), and arm length (AL) of the adults, were taken using a digital vernier caliper (accuracy: 0.05 mm). Arm length (AL) was measured using a standardized protocol based on the longest intact arm of each individual, from the arm–mantle junction to the distal tip. This criterion was selected to ensure repeatability and minimize the bias associated with partial arm loss or regeneration, which is common in octopuses. Individuals exhibiting severe arm injuries or incomplete regeneration were excluded from the analyses involving arm length. Morphometric measurements were obtained at a single time point for each individual. Repeated measurements over time were not conducted, and, therefore, the data represent a cross-sectional assessment rather than growth trajectories.

The total weight (TW) of each individual was measured using a high-precision digital scale (Ohaus^®^ Scout Pro SP 200, Parsippany, NJ, USA). The Fulton’s condition factor (*k*) was calculated as a measure of the octopuses’ health and nutritional status using the following formula:

$$k=100\times \frac{TW}{{TL}^{3}}$$

where

*k* = condition index, *TW* = total weight (g), and *TL* = total length (cm)

The condition index was used to monitor the overall physical condition of the octopuses throughout the conditioning process and to assess the effectiveness of the diet and environmental conditions on the condition index.

### 2.5. Size Regressions Analysis

To examine size-related growth patterns in *O. mimus*, linear regression models were applied to evaluate the relationships among key morphometric variables. The following regression models were fitted:

ML = a + b × TL

AL = a + b × TL

Prior to the regression analyses, all morphometric variables were natural logarithm–transformed to satisfy the assumptions of linearity and homoscedasticity, as commonly applied in cephalopod growth studies. These models provide a quantitative framework for describing size-dependent relationships and predicting growth patterns in *O. mimus*.

### 2.6. Water Quality Assessment

Throughout the study, the water quality was continuously evaluated. Parameters such as temperature, dissolved oxygen, salinity, and pH were recorded daily at two times (07:00 and 15:00 h) using a multiparameter (Brand: HACH, Loveland, CO, USA, Model: HQ4300 Multi/ISE/3 Channels) for real-time measurement. These parameters were monitored to ensure that the environmental conditions remained within the ideal range for breeding octopuses.

### 2.7. Ethical Approval of the Protocol

This study was approved by the Institutional Ethics Committee of the Jorge Basadre Grohmann National University under Constancy No. 2023-012-CEIUNJBG, in accordance with Law No. 30407 on Animal Protection and Welfare of the Ministry of Agriculture and Irrigation (MINAGRI) of Peru. All procedures for handling and treating the animals were performed in accordance with the ethical principles of animal welfare and with the due authorization of the relevant committee. After spawning and hatching, females were monitored daily. As expected for a semelparous species, post-spawning senescence was observed in spawning females. Individuals were maintained under captive conditions until the end of their natural life cycle, without euthanasia or release. Males were similarly maintained under observation after mating events and did not show immediate post-reproductive senescence during the study period.

### 2.8. Statistical Analysis

Data were analyzed using RStudio (version 2024.09.0+375; RStudio, Inc., Washington, DC, USA; source: Peru). A significance level of *p* = 0.05 was set for all the statistical tests. The Pearson correlation coefficient was used to assess the relationship between Fulton’s condition factor (*k*) and morphometric variables. Additionally, linear regressions were performed to evaluate growth, with coefficients of determination (*R*^2^) used to assess the goodness-of-fit of the models [14]. All plots were generated using the ggplot2 package in RStudio, and the results are presented as means ± standard deviation.

## 3. Results

### 3.1. Spawning

The weights of the females showed significant variability between individuals, with weights ranging from 1755 to 2977 g (Table 1). The laying dates ranged from 9 November 2023 to 16 January 2024, with an interval of 68 days between the first and last layings. The incubation period of the eggs ranged from 33 to 51 days, with the shortest incubation period recorded in a female weighing 2977 g (33 days) and the longest in a female weighing 2517 g (51 days). The temperatures during laying were 18.4–23.4 °C. The paralarvae production varied widely, from 5692 paralarvae in a female with 1905 g to 809,363 paralarvae in a female with 2769 g. The female with the highest weighting paralarvae production also recorded the shortest weighting incubation period (33 days) and the highest temperature (~23 °C).

When female size was evaluated using both total weight and mantle length, paralarvae output showed a positive but non-linear relationship, suggesting that fecundity is influenced not only by body mass but also by physiological conditions and thermal regimes during incubation.

### 3.2. Morphometric Measurement

Regression analyses performed between total length (TL) and other morphometric dimensions of *O. mimus* showed a high correlation, indicating that TL is an excellent predictor of the overall growth of octopuses. All regression models corresponded to ln-transformed morphometric variables. Specifically, the ratio of mantle length (ML) to TL showed *R*^2^ = 0.95, while the ratio of arm length (AL) to TL showed *R*^2^ = 0.96 (Table 2). The log-transformed linear regressions (Ln–Ln) revealed strong positive relationships between ML and TL, as well as between AL and TL. These relationships indicate proportional (allometric) growth patterns, whereby both mantle and arm lengths increased consistently with increasing total length across the sampled population (Table 2). The high coefficients of determination (*R*^2^) reflect a strong morphometric coupling among body dimensions.

In a complementary manner, regression analyses between length measurements and total weight (TW) demonstrated significant associations. TL was strongly correlated with total weight (*R*^2^ = 0.95, *p* < 0.001), followed by ML (*R*^2^ = 0.92, *p* < 0.01) and AL (*R*^2^ = 0.88, *p* < 0.05) (Table 3). These results indicate that, although all the morphometric dimensions evaluated are good predictors of octopus weight, total length is the most reliable indicator for estimating the biomass of individuals (Table 3).

**Figure 3:**
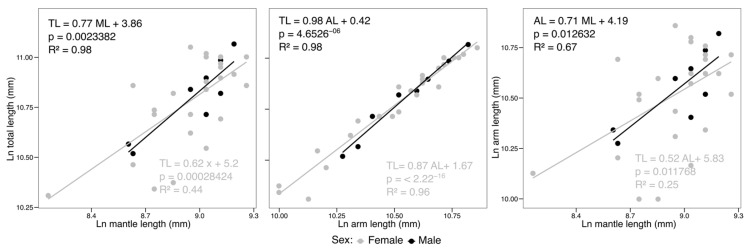
Sex-specific morphometric relationships in *Octopus mimus*. Linear regressions between mantle length and total length (ML–TL), arm length and total length (AL–TL), and mantle length and arm length (ML–AL).

Figure 3 shows the morphometric relationships between mantle length (ML), total length (TL), and arm length (AL). The data were adjusted using a linear model. For the ML-TL relationship, the lowest coefficient of determination (*R*^2^ = 0.44) was recorded in males, whereas the highest value (*R*^2^ = 0.98) was recorded in females. In both cases, a significant relationship was observed, which was stronger in females. In the AL-TL relationship, the lowest coefficient of determination (*R*^2^ = 0.96) was present in males, while the highest (*R*^2^ = 0.98) was recorded in females, showing a strong positive correlation that was significant for both sexes. For the ML-AL ratio, the lowest coefficient of determination (*R*^2^ = 0.25) was found in males, while the highest (*R*^2^ = 0.67) was found in females, indicating a moderate ratio in females and a weak ratio in males. In summary, the morphometric relationships between the variables studied were significantly stronger in females, particularly between TL-ML and TL-AL. In males, although significant relationships were also observed, the explanatory capacity of the models was weaker, particularly for the ML-AL relationship.

### 3.3. Fulton’s Condition Factor

**Figure 4:**
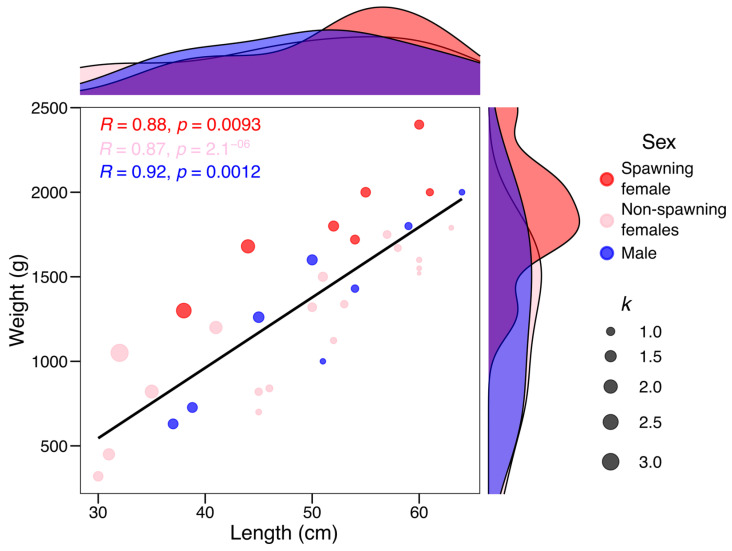
Relationship between weight and length in *Octopus mimus* across maturity stages and Fulton’s condition factor (*k*) on the Pacific Coast of Southern Peru.

Spawning females are highlighted in Figure 4 to facilitate visual comparison of *k* between spawning and non-spawning females. Spawning females tended to cluster toward higher *k* values, although no statistical comparison was performed due to the limited sample size. A significant positive correlation between weight and length was observed, with males showing a strong relationship (*R* = 0.92, *p* = 0.0012), indicating that as the length of males increases, their weight also consistently increases. In contrast, spawning females (*R* = 0.88, *p* = 0.0093) and non-spawning females (*R* = 0.87, *p* = 2.1^−6^) had a strong relationship, but weaker than that of males, reflecting greater variability in the distribution of weight and length in females. Higher values of *k* (such as *k* = 3.20 for a female weighing 1050 g and measuring 32 cm in length) indicate a better physical condition. In contrast, octopuses with low *k* values (such as *k* = 0.70 in some females) showed a lower body mass for their size. Body weight and total length distributions differed between sexes. Females exhibited a right-skewed distribution toward higher total length and body weight values compared to males (Figure 4), reflecting their larger body size at maturity. Males were generally distributed toward smaller size and weight classes.

### 3.4. Water Quality Assessment

**Figure 5:**
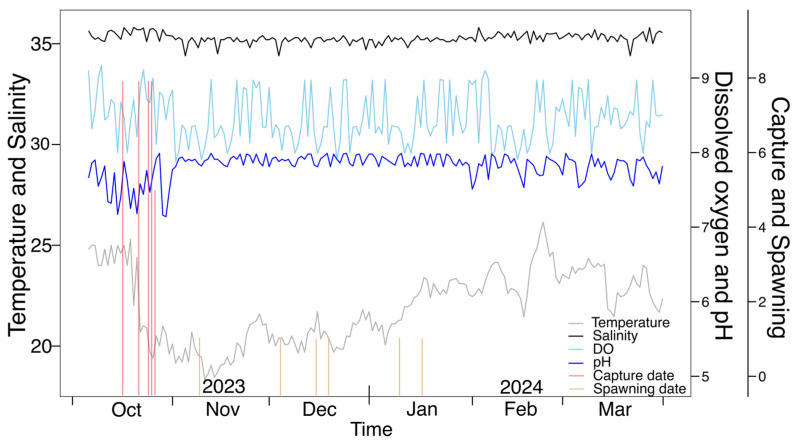
Captured specimens and spawning females of *Octopus mimus* (No. of individuals), temperature (°C), salinity (PSU), dissolved oxygen (mg/L), and pH during the evaluation.

During the conditioning process, the temperature fluctuated between 18.35 and 26.15 °C, with most values close to 21–23 °C (Figure 5). Salinity presented more pronounced variability, ranging between 34.4 and 35.81 PSU. Dissolved oxygen ranged from 7.88 mg/L to 9.17 mg/L, with most values within the range of 8.0–8.5 mg/L. The pH fluctuated between 7.14 and 8.0, with values near 7.8.

**Figure 6:**
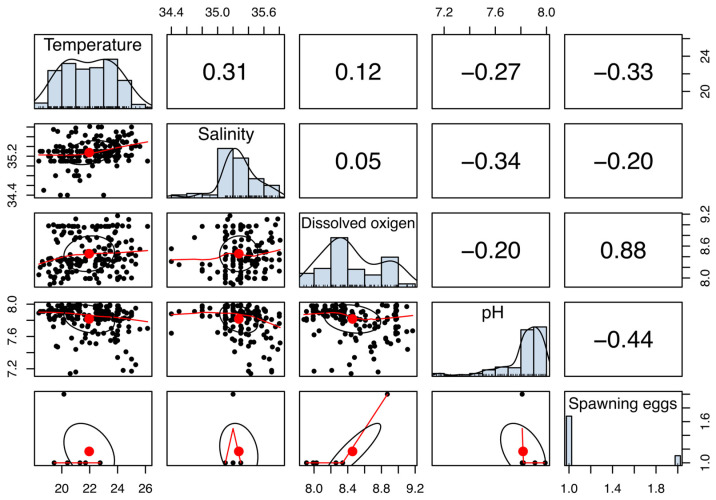
Scatter plot with distributions and multiple correlations between temperature (°C, water), salinity (PSU), dissolved oxygen (mg/L), pH and spawning occurrence in broodstock tanks of *Octopus mimus.* Red dots represent individual data points, while the red lines indicate regression lines that illustrate the relationships between parameters. The grey shaded area corresponds to the confidence intervals of the data points.

Figure 6 shows that temperature was moderately correlated with salinity (*R* = 0.31) and negatively correlated with pH (*R* = −0.44). Dissolved oxygen was strongly positively correlated with pH (*R* = 0.88) and negatively correlated with temperature (*R* = −0.20). Regarding spawning, temperature and salinity were slightly correlated with the number of spawned females, with an R value of 0.16 for salinity. There is a negative relationship with temperature (*R* = −0.18) and salinity (*R* = −0.33). Outliers in environmental variables included extreme temperatures of 18.35 °C and 26.15 °C, and dissolved oxygen of 6.5 mg/L. These conditions were included in the analysis to characterize the full range of physicochemical variability experienced by the broodstock; however, no mortality or overt stress-related behaviors were observed during the observational period.

Water quality parameters remained within relatively stable ranges throughout the conditioning and spawning periods (Table 1). No mortality or overt stress-related behaviors were observed during the observational period. The recorded environmental values, therefore, represent the physicochemical conditions under which successful spawning occurred in captivity rather than experimentally defined optimal thresholds. Exploratory correlation analyses among water quality variables revealed a significant association between pH and dissolved oxygen, consistent with expected physicochemical dynamics in marine systems. No strong correlations were detected among other parameters. These analyses were conducted to verify system stability and internal consistency rather than to infer causal relationships with reproductive performance.

## 4. Discussion

### 4.1. Spawning

Spawning in *O. mimus* is a critical aspect of aquaculture, as it determines the availability of paralarvae for growth and development in controlled systems [15]. In the results obtained in this study, it was observed that heavier females (1755 g) produced a greater number of paralarvae, which coincides with previous studies on other cephalopod species, such as *O. vulgaris* [16]. This positive correlation between female size and paralarvae production has been documented in several species, indicating that fecundity in cephalopods is closely related to body size, particularly in adult females [17,18,19,20].

The results of this study also suggest that temperature significantly affects the incubation period and egg production rate. This finding is consistent with that of Cardoso et al. [11], who reported that temperature affects both the growth rate and reproductive success of *O. mimus* and other octopus species. In this context, the optimal temperature for the reproduction of *O. mimus* should be maintained within a specific range, which in this study was 21–23 °C, similar to what was reported by other researchers [10]. Therefore, temperature control in culture systems is a crucial factor for maximizing paralarvae production and ensuring their viability in the early stages of development.

Diet quality is widely recognized as an important factor influencing growth, condition, and reproductive performance in cephalopods [12]. In the present study, a mixed diet of crustaceans, mollusks, and fish was provided to maintain broodstock under captive conditions. However, no experimental comparisons among diets, ration sizes, or feeding frequencies were conducted, and food intake was not quantified. Therefore, the present results do not allow inference on the effects of diet composition on body weight or reproductive output in *O. mimus*. Instead, the reported spawning events indicate that successful reproduction can occur under the feeding regime applied. Future studies should explicitly test dietary composition, feeding rates, and energetic intake to evaluate their effects on growth, fecundity, and paralarval quality in this species. Although larger females generally exhibited higher reproductive output, the female with the greatest body weight did not necessarily produce the highest number of paralarvae, indicating that reproductive success is modulated by multiple interacting factors, including temperature, physiological conditions, and spawning timing.

### 4.2. Morphometric Measurement

The morphometric measurements performed in this study showed a significant correlation between total length (TL), mantle length (ML), and arm length (LA), reinforcing the importance of these measurements as indicators of the growth and development of *O. mimus* in captivity. Linear regression analyses showed that total length is a good predictor of body weight in this species, a finding consistent with previous studies in other cephalopod species, such as *O. vulgaris* [7,21] and *O. maya* [22]

The relationship between TL and AL was particularly strong in smaller individual octopuses, indicating that these measurements may be useful for monitoring the growth of paralarvae and juveniles under controlled conditions. In addition, total weight (TW) measurements in relation to mantle length provide a reliable estimate of the health status of the animals, making it possible to identify potential nutritional deficiencies or growth problems in the population. These findings are consistent with reports of *O. vulgaris*, where mantle length is a good indicator of body condition [23].

Morphometric analysis of *O. mimus* also revealed that larger females tended to show a higher growth rate than males, which is consistent with what has been reported in other cephalopod studies [1]. This sexual dimorphism in growth has been widely documented in various octopus species, where females tend to reach larger sizes and have a greater reproductive capacity [7,11,21]. This pattern also has important implications for aquaculture, as larger females can be selected as breeders to optimize paralarvae production in culture systems. Morphometric data were collected at a single time point; the present results do not allow inference on individual growth rates or developmental changes over time. Instead, the observed morphometric relationships describe size structure and body condition at the time of reproduction under captive conditions. Future studies should incorporate longitudinal designs with repeated measurements to evaluate growth dynamics and their influence on reproductive performance in *O. mimus*.

### 4.3. Fulton’s Condition Factor

The *k* is a widely used index to describe the overall body condition and energetic status of aquatic organisms, including cephalopods [24]. In the present study, *k* was calculated using body weight and mantle length, providing a snapshot of individual condition at the time of spawning under captive conditions.

Females exhibiting higher *k* values also showed higher egg production, indicating a positive association between body condition and reproductive output. Similar relationships between female size, energetic reserves, and fecundity have been reported in other octopod species, suggesting that individuals in better physiological condition are able to allocate more energy to reproduction [2,6].

However, it is important to note that growth rates over time, dietary intake, and paralarval survival were not experimentally assessed in this study. Therefore, the observed associations between *k* and reproductive output should not be interpreted as causal relationships. Instead, the results indicate that successful spawning occurred under captive conditions where broodstock maintained relatively high condition indices.

Previous studies have highlighted the importance of diet quality, water quality, and temperature in influencing body condition and reproductive performance in octopus aquaculture [25,26,27,28]. Future experimental studies incorporating controlled feeding regimes, longitudinal growth measurements, and assessments of paralarval survival will be necessary to determine how variation in condition factor directly influences reproductive efficiency in *O. mimus* aquaculture.

### 4.4. Water Quality Assessment

Water quality is one of the most critical factors in octopus aquaculture, as it directly influences the growth, reproduction, and overall health of the octopuses [29]. In this study, various water quality parameters were monitored, including temperature, salinity, pH, and dissolved oxygen, which were adjusted to create an optimal environment for octopus culture. It is important to emphasize that the present study was observational and did not aim to experimentally optimize environmental conditions for reproduction. Consequently, the water quality parameters reported here should be interpreted as baseline conditions associated with successful spawning rather than as predictors of reproductive performance or survival. No experimental manipulation of environmental variables, behavioral monitoring, or mortality-based endpoints was conducted.

These findings are consistent with those of other studies on octopus and related species, such as *O. vulgaris*, where variability in water quality parameters has been shown to affect octopus growth rate and survival [8,17]. In particular, temperature has a crucial impact on the incubation period and the development of paralarvae [30,31], as observed in the results of this study. Efficient water quality management in recirculating systems (RAS) is therefore essential to maximize the productivity and sustainability of *O. mimus* aquaculture [2,32].

Research on water quality in cephalopod aquaculture systems has also pointed to the importance of maintaining a proper balance between nutrients and oxygenation, as high ammonia concentrations and lack of oxygen can have negative effects on animal welfare [11]. This study reinforces the need to implement filtration and recirculation systems that maintain optimal water quality to ensure a high growth rate and successful reproduction in aquaculture systems. The present study does not define tolerance limits of environmental variables but rather reports the range of conditions under which successful spawning occurred. Future studies should incorporate comprehensive water chemistry monitoring, including nitrogenous waste compounds.

### 4.5. Implications for Aquaculture

The results of this study provide key information for the management of *O. mimus* in aquaculture systems. Controlling temperature [26,27,33], salinity, and dissolved oxygen [29] is critical for optimizing octopus’s reproduction and growth. In addition, the positive correlation between female size and paralarvae production suggests that selecting larger females for breeding may improve octopus production efficiency [1]. This is in line with the recommendations of Boletzky [34] and other researchers who have highlighted the importance of selecting broodstock based on their size and condition to maximize aquaculture reproductive success. The use of predictive models based on the total length of octopuses is also a useful tool for aquaculturists, as it allows for the estimation of biomass and planning production efficiently [24]. These models provide a more accurate approach to crop management, reducing uncertainty and improving the economic sustainability of aquaculture. From a macroevolutionary perspective, cephalopods exhibit trade-offs between growth rate, fecundity, and lifespan, which shape their reproductive strategies [35]. The successful reproduction of *O. mimus* under controlled conditions aligns with broader life-history trends described for cephalopods, reinforcing its suitability for aquaculture diversification.

Although this study provides valuable insights into the reproductive biology and management of *O. mimus*, some areas require further investigation [17]. In particular, the impact of genetic factors on the reproductive and growth variability of octopuses should be investigated. It is also necessary to investigate the interactions of other environmental factors, such as luminosity and photoperiod, on the physiology of octopuses in culture systems [36]. Improved diets and management of octopuses could contribute significantly to increasing reproductive efficiency and reducing the operating costs of octopus aquaculture [37].

## 5. Conclusions

The evidence obtained shows that *O. mimus* can reproduce successfully under environmental conditions in southern Peru, establishing a technical framework for its aquaculture management. It was confirmed that the size of females is a determining factor in paralarvae production, supporting the need to select broodstock with higher biomass to optimise reproductive efficiency. Likewise, temperature significantly influenced the duration of the incubation period, with processes between 22 and 23 °C being the most efficient. Morphometric relationships showed robust correlations between total length, mantle and arms, allowing the generation of predictive models useful for estimating growth and body condition. The Fulton index showed physiological variability associated with the nutritional status of the organisms. Finally, water quality parameters remained within optimal ranges, demonstrating that strict environmental control is essential to ensure the welfare, reproduction and viability of *O. mimus* in farming systems. Given its merobenthic life cycle, commercial importance, and demonstrated reproductive capacity under controlled conditions, *O. mimus* represents a promising candidate not only for commercial aquaculture but also for restorative aquaculture initiatives aimed at reducing fishing pressure on natural populations.

## Figures and Tables

**Table 1:** Summary of reproductive performance in *Octopus mimus* females: weight, spawning and hatching dates, incubation period, temperature, and paralarvae production.

Female	Weight (g)	Mantle Length (mm)	Spawning Date	Embryo Incubation Time (Days)	Hatching Date		Hatching (Days)	Spawning Temperature (°C)	No. of Paralarvae
					Beginning	Final			
1	2517	151	9 November 2023	51	30 December 2023	10 January 2024	12	18.4–20.2	Undeterminated
2	2332	15.0	4 December 2023	47	19 January 2024	29 January 2024	11	19.7–20.9	121,489
3	1905	12.0	15 December 2023	46	29 January 2024	2 February 2024	5	20.2–21.7	5692
4	2765	16.2	19 December 2023	44	31 January 2024	9 February 2024	10	19.7–21.3	366,063
5	1755	135	19 December 2023	44	31 January 2024	1 February 2024	2	19.7–20.2	7022
6	2769	16.0	10 January 2024	38	16 February 2024	25 February 2024	10	21.3–23.4	809,363
7	2977	16.3	16 January 2024	33	17 February 2024	27 February 2024	9	22.6–23.4	784,775

**Table 2:** Morphometric regression equations for *Octopus mimus*.

Morphometric Relationship	Regression Equation	*R* ^2^
Ln (ML) vs. Ln (TL)	Ln (ML) = 0.67 + 0.77 · Ln (TL)	0.50
Ln (AL) vs. Ln (TL)	Ln (AL) = −1.17 + 1.08 · Ln (TL)	0.96

**Table 3:** Results of regression analyses between different measurements of length and weight of *Octopus mimus*.

Independent Variable	Dependent Variable	*R* ^2^	*p*-Value
TL	Total weight (TW)	0.95	<0.001
ML	Total weight (TW)	0.92	<0.01
AL	Total weight (TW)	0.88	<0.05

## Data Availability

The data presented in this study are available on request from the corresponding author.
